# The Orphanet Nomenclature and Classification of Rare Diseases for Improved Patient Recognition and Data Interoperability: Qualitative and Quantitative Analysis

**DOI:** 10.2196/84553

**Published:** 2026-07-08

**Authors:** Caterina Lucano, David Lagorce, Annie Olry, Houda Ali, Valérie Lanneau, Mickael De Carvalho, Aysegul Dilsizoglu Senol, Marta Fructuoso, Emilie Gaillard, Marie-Cécile Gaillard, Seed Mihic, Mariana Tannoury, Florence Sauvage, Charlotte Rodwell, Sylvie Maiella, Marc Hanauer, Ana Rath

**Affiliations:** 1 US14-Orphanet Inserm Paris, Île-de-France France; 2 Banque Nationale de Données Maladies Rares (BNDMR) Assistance Publique – Hôpitaux de Paris Paris, Île-de-France France

**Keywords:** classification, codification, Interoperability, medical terminology, nomenclature, ORPHAcodes, rare diseases

## Abstract

**Background:**

Although individually uncommon, rare diseases (RDs) collectively affect an estimated 329-624 million people worldwide. There are over 6500 known RDs, 85% of which affect fewer than 1 person per million. Consequently, the critical amount of data necessary to improve knowledge, care, and treatment can only be achieved through cumulative data collection across countries. However, RDs remain underrepresented in medical terminologies and classification systems, hindering data sharing, interoperability, and public health monitoring.

**Objective:**

This paper presents the Orphanet Nomenclature and Classification of RDs detailing its content, production and update methodology, and mappings to other semantic resources. It also provides an up-to-date count of RDs based on the consensus operational definition describing their distribution by medical domain.

**Methods:**

The Orphanet Nomenclature of RDs is a multilingual standardized system composed of clinical entities, each defined by a unique and time-stable ORPHAcode, a preferred term, synonyms, a classification level, and a textual definition. This nomenclature is structured into 3 classification levels organized within a multihierarchical and multiparental classification system by medical domain. Its production, updates, and mappings to major biomedical resources rely on standardized and published procedures, continuous literature review, manual curation, and expert validation, reflecting advancements in RDs knowledge and clinical practice. Presented data metrics were computed using the Orphanet July 2025 release to quantitatively characterize the content, structure, classification, and semantic alignments of the Orphanet Nomenclature and Classification system.

**Results:**

As of July 2025, the Orphanet Nomenclature of RDs includes a total of 9784 active clinical entities, including 6527 disorders (corresponding to the RDs definition), 1084 subtypes of disorders, and 2173 groups of disorders. Disorders are multiclassified into 29 classification hierarchies, each corresponding to a distinct medical domain, accurately representing the complex multisystemic nature of RDs. Extensive qualified mappings ensure semantic interoperability: 97.4% (6355/6527) of disorders are mapped to at least 1 *ICD-10* (*International Statistical Classification of Diseases, Tenth Revision*) code (415/6527, 6.4% with an exact proximity relationship), 71.8% (4683/6527) are mapped to at least 1 *ICD-11* (*International Classification of Diseases, Eleventh Revision*) Mortality and Morbidity Statistics code (958/6527, 14.7% with an exact relationship) and 94.8% (6191/6527) are mapped to Systematized Nomenclature of Medicine Clinical Terms (all with an exact relationship). Genetic disorders represent 72.2% (4715/6527) of all RDs, and 63.4% (4141/6527) are mapped to at least 1 phenotypic Online Mendelian Inheritance in Man number.

**Conclusions:**

The Orphanet Nomenclature and Classification of RDs is the only RDs-specific interoperable medical terminology meeting the needs of health care, research, and public health systems. By addressing the underrepresentation of RDs in medical terminologies, it enables accurate RDs identification, coding, and monitoring, supporting cross-border data interoperability, and contributing to improved knowledge, policymaking, and ultimately better care for people living with an RD.

## Introduction

Although rare diseases (RDs) are individually uncommon, with over 6500 different known RDs, they collectively affect an estimated 329-624 million people worldwide [1,2]. Despite this significant global burden, available data indicate that 85% of RDs affect less than 1 person per million [1]. As a result, the critical amount of data necessary to improve knowledge, care, and treatment can only be achieved through cumulative data collection across different countries in a standardized manner [3,4]. Cross-border data collection, sharing, and exploitation to support evidence-based policymaking, health care, and research are therefore essential in the field of RDs, as underscored by numerous international and European initiatives [5].

Over the past decades, numerous definitions of RDs have been proposed, reflecting local legislations and public health priorities [4,6-11], most of which use point prevalence as the common epidemiological indicator. However, the threshold for rarity varies geographically, ranging from less than 1 per 2500 in Japan [12] to no more than 50 per 100,000 in Europe [13], or no more than 200,000 persons in the United States [14]. Furthermore, definitions can differ clinically, sometimes including restrictions such as “life-threatening” or “chronically debilitating” [6,13,15]. To address these inconsistencies, Rare Diseases International (RDI), in collaboration with a global panel of experts and the World Health Organization (WHO), has developed an operational description of rare diseases, defining an RD as a “medical condition with a specific pattern of clinical signs, symptoms, and findings that affect fewer than or equal to 1 in 2000 persons living in any WHO-defined region of the world” [16]. Despite these efforts, the lack of harmonization among countries continues to impact RDs research, diagnosis, and health care planning [3,5,8].

To take public health action on RDs, it is fundamental to measure how many people are affected by these diseases in a defined population (prevalence), how these conditions impact those affected, and how to monitor their medical and societal consequences. [1,4,17]. In this regard, the United Nations resolution on people living with an RD “encourages Member States and relevant United Nations agencies to collect, analyze, and disseminate disaggregated data on persons living with a rare disease... to assess progress towards the improvement of the status of persons living with a rare disease.” This further highlights that adequate coding of data of patients with RD within health information systems is a prerequisite for an effective response to this global public health priority [18].

However, RDs are underrepresented in medical terminologies and classification systems used within health systems, with only a small fraction of RDs possessing specific and unambiguous codes [19-22]. This limitation makes it difficult to trace patients with RD in health information systems, preventing a robust understanding of the actual number of people living with RD. Only a small proportion of RDs have a specific code in the *ICD-10* (*International Statistical Classification of Diseases, Tenth Revision*) [20-22], the most widely used codification system worldwide; the representation of RDs has increased in the *ICD-11* (*International Classification of Diseases, Eleventh Revision*) [21,23] and the Systematized Nomenclature of Medicine Clinical Terms (SNOMED CT) [24], though it still remains below the number of known RDs. Moreover, RDs cannot be easily identified in these reference systems, as they do not explicitly include any indication of the rarity of a disease. Finally, different regions use diverse semantic resources in their coding systems (eg, *ICD-10* or SNOMED CT), or diverse versions of the same resource (*ICD* [*International Classification of Diseases*] local extensions or different *ICD* versions), hampering data sharing and interoperability. Given the already scarce nature of RDs data, this barrier to data collection requires dedicated efforts to make RDs recognizable in health information systems [25-27].

To tackle the challenges of RDs codification and interoperability, Orphanet has developed and maintained an inventory of RDs since 1997 and a nomenclature and classification of RDs since 2007 [20,28]. Over time, adoption of the Orphanet Nomenclature of RDs for RDs codification within national health systems has been supported and endorsed by European and international policy initiatives aimed at improving RDs identification and monitoring and RDs cross-border data sharing. The Orphanet Nomenclature and Classification of RDs is now recognized as the reference resource to support standardized RDs codification across health care and research settings.

The aim of this paper is to present the Orphanet Nomenclature and Classification of RDs, detailing its content as well as its production and update methodology, and an overview of its mappings to other medical terminologies and classification systems. Additionally, it provides a clear and up-to-date count of RDs based on the above-mentioned consensus definition, and their distribution by medical domain. Finally, it discusses the importance of maintaining and adopting a standardized and reliable RDs nomenclature and classification system to support clinical coding, research, health care interoperability, and policymaking.

## Methods

### Structural Description of the Orphanet Nomenclature and Classification of RDs

#### The Core Elements of the Orphanet Nomenclature and Classification of RDs

The Orphanet Nomenclature of RDs is a unique and multilingual standardized system aimed at providing a specific reference standard for RDs, which is organized in a classification system, a multihierarchical and polyparental structure organized by medical domain according to diagnostic and therapeutic relevance. The Orphanet Nomenclature of RDs is composed of clinical entities (a generic technical term used to describe the clinical items included in the nomenclature), each of which includes several elements to ensure precise and unambiguous representation of RDs concepts (Textbox 1) [29]:

- ORPHAcode: a unique and time-stable numerical identifier automatically assigned in ascending order to every entry registered in the Orphanet Nomenclature.
- Preferred term (or main name): the most widely accepted name for the diagnosis in the literature and/or in the medical community.
- Synonyms: additional terms, including acronyms, fully equivalent to the preferred term, as many as necessary according to the information found in the literature.
- Classification level: indicates the level of precision of the clinical entity, among 3 possible granularity levels (group of disorders, disorder, or subtype of disorder).
- Definition: a brief description of the major clinical characteristics that define the diagnosis and differentiate it from other related clinical entities.

**Technical elements composing the Orphanet Nomenclature of rare diseases. Example of a clinical entity in the Orphanet Nomenclature of Rare Diseases, defined by an ORPHAcode, a preferred term, as many synonyms as needed, a classification level, and a textual definition.**

**ORPHAcode**

- ORPHA:2134

**Preferred term**

- Atypical hemolytic uremic syndrome

**Synonyms**

- Atypical HUS; aHUS

**Classification level**

- Disorder

**Definition**

- A rare, genetic thrombotic microangiopathy due to dysregulation of the alternative complement pathway and characterized by the triad of hemolytic anemia, thrombocytopenia, and acute renal dysfunction.

#### Naming Rules and Multilingual Translation of the Orphanet Nomenclature of RDs

In the absence of an international consensus on how to name diseases, preferred terms and synonyms are assigned according to standard naming rules developed by Orphanet to create a comprehensive RDs nomenclature that aligns with current knowledge and clinical practice [30]. These rules ensure consistency across different disorder groups and medical domains, facilitating better integration and use of RDs-related clinical information in databases and health care systems. The Orphanet Nomenclature of RDs is produced in English and entirely translated into 8 languages: French, German, Spanish, Italian, Dutch, Portuguese, Polish, and Czech, by translation teams located in member countries of the Orphanet Network. All translations are validated by RDs expert medical doctors in each country. The English nomenclature serves as the basis for the translation of concepts; however, the translation process considers the particularities and local practices specific to every language, in particular with regard to the number of synonyms [31].

#### Classification Levels and Typology

**Figure 1:**
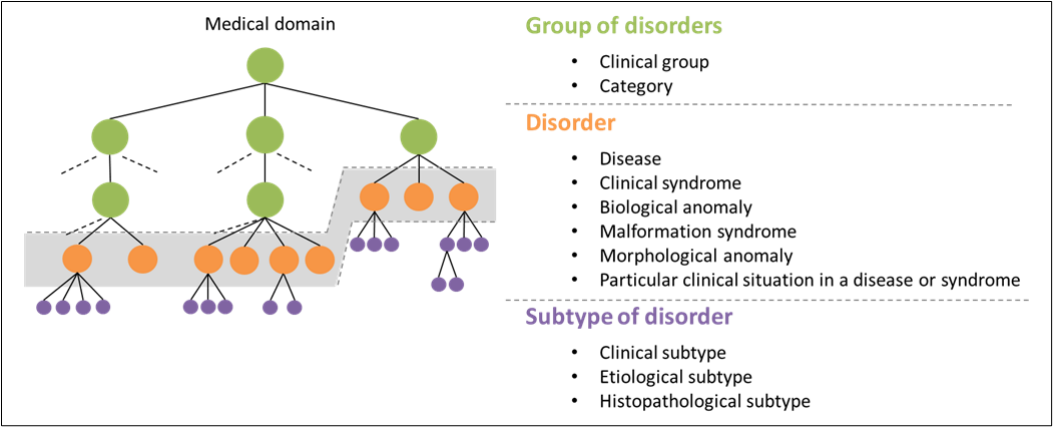
Classification levels and typologies of the Orphanet Nomenclature and Classification of rare diseases. Every clinical entity in the Orphanet Nomenclature and Classification of rare diseases is associated with a classification level conveying the precision of the diagnosis (group of disorder, disorder, or subtype of disorder), and a unique typology within the classification level.

The Orphanet classification system is composed of the 3 above-mentioned classification levels, namely group of disorders, disorder, and subtype of disorder (Figure 1). The classification level serves as the basis for parent-child relationships between related clinical entities. These relationships structure the Orphanet classification according to successive criteria that are relevant in the clinical setting [29]. Additionally, each clinical entity is attributed a typology, a characteristic that, within the classification level, conveys the nosological definition of the clinical entity. For a group of disorders, typologies include clinical group and category; for disorders, they include disease, clinical syndrome, biological anomaly, malformation syndrome, morphological anomaly, and particular clinical situation in a disease or syndrome; and for subtype of disorder, they include clinical subtype, etiological subtype, and histopathological subtype (Figure 1; definitions in Multimedia Appendix 1) [29]. Of note, while generally used interchangeably to accommodate the widespread use of the term “rare diseases,” the terms “disease” and “disorder” designate different concepts within the Orphanet Nomenclature of RDs. The term “disorder” is used to refer to the classification level that includes the 6 different typologies mentioned above, while the term “disease” represents a specific typology and a narrower clinical concept within the “disorder” classification level (Figure 1; Multimedia Appendix 1) [29]. In the Orphanet Nomenclature and Classification system, the “disorder” level corresponds to the WHO-RDI consensus definition of “rare disease” [16].

### Production and Update Process of the Orphanet Nomenclature and Classification of RDs

#### Curation and Update Procedure of the Orphanet Nomenclature and Classification of RDs

The Orphanet Nomenclature and Classification of RDs is produced and updated according to a standardized and published procedure, in order to maintain an optimal quality of information, by a scientific team of information scientists at the Institut national de la santé et de la recherche médicale (INSERM) US14 service unit, Paris, France. The information provided in the Orphanet Nomenclature is sourced from up-to-date scientific and medical literature (more than 2000 papers are consulted yearly), manually curated, reviewed, and validated by Orphanet information scientists and RDs medical experts [29,32].

The maintenance of the Orphanet Nomenclature of RDs is ensured through 3 main types of actions:

- Inclusion of a new clinical entity: to be included in the Orphanet Nomenclature, a disorder-level clinical entity must correspond to a well-delineated clinical presentation, have a prevalence of fewer than or equal to 1 in 2000 persons, and be described in at least 2 unrelated individuals in the literature, or 2 individuals from the same family when there is a clear genetic cause identified. Groups of disorders and subtypes of disorders are included according to established clinical practice and evolving medical knowledge. Before inclusion, the clinical entity is compared with existing entities to avoid overlaps and ensure classification consistency.
- Modification of an existing entity: a clinical entity in the Orphanet Nomenclature of RDs can be modified to improve or update its information, including the preferred term, synonyms, classification level, typology, or definition. Clinical entities described before the genetic era, and lacking publications since then, are labeled as “historic” and are kept in the Orphanet Nomenclature because their initial descriptions are still available and carry a potential for further characterization.
- Inactivation of a clinical entity: a clinical entity in Orphanet can be inactivated due to deprecation (it is now included in another concept), obsolescence (it has no valid reason to exist, such as being an imprecise or duplicate concept), or for no longer meeting the rare disorder prevalence criteria.

#### Management of Inactivation, Reactivation, and Replacement of ORPHAcodes

An ORPHAcode is permanently linked to the same clinical concept and is never deleted or reused for a different entity, even if the original entity becomes inactive. In rare instances, an inactive clinical entity may be reactivated after reexamination with updated knowledge and reintroduced into the Orphanet Nomenclature and Classification with all relevant scientific information. To ensure continuity of information, especially in patient records and coding systems, Orphanet provides a replacement active clinical entity for deprecated and obsolete entities. This replacement code, or target code, must be used instead of the corresponding deprecated clinical entity, as it aligns with the evolution of knowledge in the literature. For obsolete entities, the target often refers to a group of disorders, guiding users to explore the relevant classification to identify the most appropriate replacement code based on the patient’s diagnosis. Entities inactivated as nonrare in Europe are not given a replacement code, as they no longer fall under the RDs scope.

### Classification of RDs by Medical Domain

#### The Orphanet Classification Hierarchies

The Orphanet classification is structured in 34 major classification groups, individually referred to as the classification hierarchy. Of these, 29 represent a distinct medical domain and are listed in Multimedia Appendix 2. The remaining 5 hierarchies are ad hoc classifications designed primarily for organizational purposes:

- Rare genetic diseases: this classification does not represent a distinct medical domain but is rather a derived genetic version that mirrors all the other clinical classification hierarchies.
- Rare systemic and rheumatological diseases of childhood: this classification is fully included in the Orphanet classification of rare systemic or rheumatologic disorders.
- Rare transplant-related diseases: this classification does not represent a medical domain but is used to classify disorders according to an intervention.
- Rare teratologic disorders: this classification is fully included in the Orphanet classification of rare developmental anomalies during embryogenesis.
- Rare disorder without a determined diagnosis after full investigation: this classification does not represent a medical domain and is used to classify a single disorder, ORPHA:616874 rare disorder, without a determined diagnosis after full investigation [33]. This is a code that does not conceptually fall under any of the other Orphanet classification hierarchies, as it allows for the identification in health care information systems of patients who remain undiagnosed after a full investigation.

Every classification consists of a tree structure starting with a unique ultimate parent group at the top representing the broadest clinical concept/medical domain (head of classification), then branching out into as many intermediate groups as necessary with increasing precision of clinical concepts, until reaching the first level of the confirmed diagnosis, the disorder. For any given disorder, subtypes can be added when this level of precision is required in clinical practice for appropriate management and follow-up.

#### Multihierarchical Classification and Linearization

**Figure 2:**
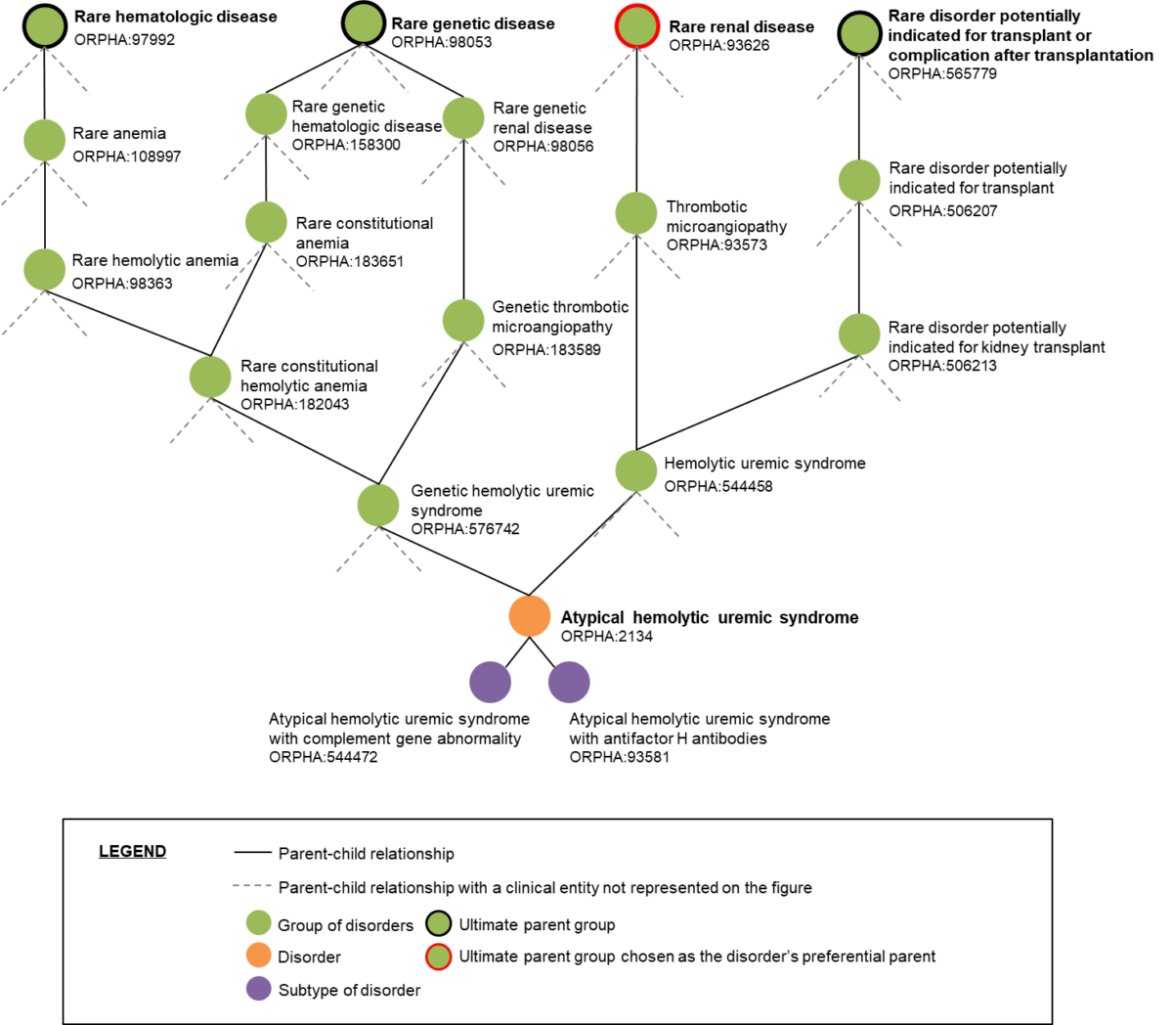
Representation of a disorder in the Orphanet classification system. The disorder Atypical hemolytic syndrome (ORPHA:2134) is classified into 4 classification hierarchies. It has 2 direct parent groups, each with broader parent groups, culminating at the top of the hierarchy, where each ultimate parent group represents a medical domain. The rare renal disorder group (ORPHA:93626) was designated as the preferential parent of the disorder Atypical hemolytic syndrome (ORPHA:2134). Note that all groups in the figure have additional child entities not shown here.

Every clinical entity in the Orphanet Nomenclature of RDs is introduced in as many classification hierarchies as necessary according to major medical domains involved in the management of the disorder. This results in a multihierarchical and multiparental classification structure that reflects the multidimensionality and complexity of RDs (Figure 2). However, only 1 ultimate parent group is chosen as the entity’s preferential classification (preferential parent), according to the most severely affected body system, the most determining involvement for the prognosis, and/or the medical domain that will most likely be relied on for the management of the disorder. This process, named linearization, enables coherent statistical analysis. For a substantial number of RDs, no firm scientific basis exists to support assignment to a single medical domain, making preferential parent selection inherently complex. Linearization in these situations is a conventional framework guided by a set of published rules aimed at limiting arbitrariness and ensuring consistent application [34].

#### Mappings to Other Semantic Resources

The Orphanet Nomenclature of RDs is mapped to other international terminologies, classification systems, controlled vocabularies, knowledge bases, and ontologies according to a standardized and published procedure, to enable interoperability between different information systems [35]. Target standards include the WHO classification systems *ICD-10* [36] and *ICD-11*, both Mortality and Morbidity Statistics (MMS) and Foundation [37], the clinical terminology SNOMED CT [38,39], the Online Mendelian Inheritance in Man (OMIM) knowledge base [40], the metathesaurus Unified Medical Language System (UMLS) [41], the controlled vocabularies Medical Subject Headings (MeSH) [42] and Medical Dictionary for Regulatory Activities (MedDRA) [43], the NIH (National Institutes of Health), the Genetic and Rare Diseases Information Center (GARD) knowledge base [44], and the Mondo Disease Ontology (Mondo) [45].

The mapping cardinality always goes from the ORPHAcode to a code of the target resource. All mappings are qualified by a proximity relationship that defines the nature of the correspondence between the two concepts. The adopted proximity relationships are as follows:

- Exact: the Orphanet entity designated by an ORPHAcode and the target code have the same range of application; they describe the same pathological entity.
- Broader term to narrow term: the Orphanet entity designated by an ORPHAcode has a broader range than the target code.
- Narrow term to broader term: the Orphanet entity designated by an ORPHAcode has a narrower range than the target code.

Mappings are generally validated at the Orphanet disorder and subtype levels; however, when an exact corresponding concept exists in the target resource, mappings may also be retained at the group level. For *ICD-10*, *ICD-11,* and OMIM, mappings are maintained with all 3 possible proximity relationships, whereas for SNOMED CT, UMLS, MeSH, MedDRA, GARD, and Mondo, only exact matches are retained.

The mappings with the WHO *ICD-10* and *ICD-11* are further characterized by a specificity relationship, describing how the equivalent clinical concept is represented in *ICD* [35]. The relationships are defined as follows:

- Specific code: the clinical concept represented by the ORPHAcode corresponds to a code in the *ICD-10* tabular list/*ICD-11* MMS exclusively denoting the concept.
- Index term: the clinical concept represented by the ORPHAcode is listed as an entry term in the *ICD-10* alphabetical index or in the *ICD-11* Foundation, without being assigned a dedicated tabular list/MMS code.
- Inclusion term (*ICD-10* only): the clinical concept represented by the ORPHAcode appears as an inclusion term under an *ICD-10* term in the tabular list without a distinct code.
- Attributed code: in the absence of an explicit corresponding term in *ICD-10* or *ICD-11* for the clinical concept represented by the ORPHAcode, an *ICD* code is attributed by Orphanet as the closest available clinical concept.

All mappings are manually curated and validated by an Orphanet information scientist and a medical doctor before publication, unless explicitly marked as *Not yet validated* to indicate pending medical review. Mappings with SNOMED CT concepts result from a collaborative process with SNOMED International, in which Orphanet disorder concepts are first evaluated for inclusion in SNOMED CT by a SNOMED International terminologist, and the resulting exact mappings are subsequently validated jointly by SNOMED International and Orphanet before publication. Notably, only mappings to *ICD-10*, *ICD-11,* and OMIM are distributed together with the official Orphanet Nomenclature files for coding, while the entirety of the mappings is distributed through Orphadata Science.

### Data Extraction and Analysis

#### Datasets Description

Data metrics were computed using the Orphanet Scientific Knowledge Files delivered on the Orphadata Science website [46]. These datasets, structured in an XML file format and in JSON format, are released in July every year at the same time as the official Orphanet Nomenclature files for coding (Nomenclature Pack) [47]. For this study, we analyzed the following datasets of the July 2025 release:

- the “rare diseases and alignments with terminologies and databases” dataset (<iso>_product1.xml) in English, Czech, Dutch, French, German, Italian, Polish, Portuguese, and Spanish languages; version 1.3.42/4.1.8 [3] released 24 Jun 2025.
- the “linearization of rare diseases” English dataset (en_product7.xml); version 1.3.42/4.1.8 [3] released 24 Jun 2025.
- the “genes associated with rare diseases” English dataset (en_product6.xml); version 1.3.42/4.1.8 [3] released 24 Jun 2025.
- The “classifications of rare diseases” English datasets (en_product3_<x>.xml); version 1.3.42/4.1.8 [3] released 24 Jun 2025.

Moreover, the differential files of the Orphanet Nomenclature packs for coding (ORPHAnomenclature_diff_en.xlsx) released from 2020 to 2025 were used to manually retrieve the annual inclusion and inactivation of disorders, and the human-readable Orphanet-SNOMED CT mapping file (ORPHA-SNOMEDCT_Mapping_File_production.xlsx), July 2025 version, released in October 2025, was used to report on the Orphanet-SNOMED CT mappings.

#### Metrics Calculation

Metrics were computed using Python 3.8 scripts made available as dedicated Jupyter Notebooks (.ipynb files) in a Binder/Github-ready repository [48]. This repository provides a publicly accessible, reproducible, and interactive computational environment that enables on-demand calculation of metrics using Orphadata files. A total of 8 scripts were developed to calculate the necessary metrics and were annotated to document the calculation methodology. Metrics not computable via scripting were calculated manually, as detailed below.

The distribution of active clinical entities by classification level and typology was calculated using the script “1_number_of_Orphanet_clinical_entities.” This script uses the “rare diseases and alignments with terminologies and databases” dataset in English, filters active and inactive clinical entities, and subsequently counts them by classification level and typology. The same dataset was used to manually calculate the number of historical clinical entities by filtering for the “historical entity” annotation.

The distribution of synonyms by classification level in English, as well as the number of adapted synonyms in Czech, Dutch, French, German, Italian, Polish, Portuguese, and Spanish, was calculated with the script “2_production_of_the_­Orphanet_RD_terminology_in_english_and_other_language.” This script uses the “rare diseases and alignments with terminologies and databases” dataset in all target languages and sequentially filters and counts annotations for active clinical entities, and retrieves and counts their synonyms, grouping them by classification level.

The number of translated preferred terms in Czech, Dutch, French, German, Italian, Polish, Portuguese, and Spanish was calculated manually by comparing the preferred terms of active clinical entities in each target language with the corresponding English preferred terms in the “rare diseases and alignments with terminologies and databases.” Terms differing from the corresponding English term were considered translated; terms identical to the corresponding English term were considered untranslated, with the exception of acronyms and Latin terms, which were considered translated.

The coverage of definitions at the disorder level in each language was calculated using the script “8_number_of_Orphanet_RD_with_definition_in_all_languages.” This script uses the “rare diseases and alignments with terminologies and databases” dataset in all target languages, filters active clinical entities at the disorder classification level, and retrieves and counts the corresponding definitions.

The annual disorder inclusions and inactivations for the period 2020-2025 were manually retrieved from the differential files of the Orphanet Nomenclature packs for coding released between 2020 and 2025.

The number of disorders classified in more than one classification hierarchy vs those classified in a single classification hierarchy was calculated using the script “4_number_of_Orphanet_RD_monoclassified_vs_multiclassified” which uses the “classifications of rare diseases” datasets to select disorders and assess their inclusion in one or multiple hierarchies. The same datasets are exploited by the script “5_distribution_of_Orphanet_RD_in_Orphanet_classifications” to retrieve and count the number of disorders included in each classification hierarchy. Of the 34 classification hierarchies in the Orphanet classification system, only 29 were included in the analysis. The previously described 5 hierarchies not corresponding to distinct medical domains or representing organizational or nonclinical groupings were excluded. The distribution of disorders by preferential parent was calculated using the script “6_distribution_of_ Orphanet_RD_by_preferential_parent.” This script selects all active disorders from the “rare diseases and alignments with terminologies and databases” dataset and uses the “linearization of rare diseases” dataset to retrieve their preferential parent, subsequently counting the total number of disorders linearized for each classification hierarchy.

The proportion of genetic disorders was calculated using the script “3_proportion_of_genetic_versus_non_genetic_ Orphanet_RD.” This script retrieves all active disorders from the “rare diseases and alignments with terminologies and databases” dataset and assesses their inclusion in the “genes associated with rare diseases” dataset and the “classifications of rare diseases-rare genetic diseases classification” dataset. Inclusion in either dataset was considered indicative of a genetic disorder.

Mappings to other semantic standards were retrieved using the script “7_alignment_between_Orphanet_RD_and_international _terminologies” for *ICD-10*, *ICD-11*, and OMIM, and manually for UMLS, MeSH, MedDRA, GARD, and Mondo. In both cases, the “rare diseases and alignments with terminologies and databases” dataset was filtered by the target standard to retrieve mappings at the Orphanet disorder level and their relationships. Orphanet-SNOMED CT mappings were retrieved manually by filtering the human-readable Orphanet-SNOMED CT mapping file for mappings at the disorder level.

### Ethical Considerations

This study did not involve human participants, patient-level data, or identifiable personal information. The analyses were conducted exclusively using data derived from published literature and publicly available sources curated within Orphanet. Consequently, approval from an institutional review board or equivalent ethics committee was not required, and informed consent was not applicable.

## Results

### Quantitative Characterization of the Orphanet Nomenclature and Classification of RDs

#### Counting and Distribution of Active Clinical Entities in the Orphanet Nomenclature and Classification of RDs by Classification Level and Typology

As of July 2025, the Orphanet Nomenclature of RDs contains a total of 9784 active clinical entities, including 6527 disorders corresponding to the consensus definition of “rare disease” [16] (among which is a specific disorder code for undiagnosed patients after full investigation [33]), 1084 subtypes of disorders, and 2173 groups of disorders. Among all active entities, 3.3% (325 disorders and 1 subtype) are considered historic, with no publications over the past 25 years. The distribution of all active clinical entities by their classification level and their typology is presented in Table 1 and in Multimedia Appendix 3. Definitions of each typology are provided in Multimedia Appendix 1.

**Table 1:** Distribution of active clinical entities in the Orphanet Nomenclature of Rare Diseases by classification level and typology.

Classification levels		Active clinical entities, n (%)^a^
**Disorders**		6527 (66.7)
	Diseases	4172 (63.9)
	Clinical syndromes	50 (0.8)
	Malformation syndromes	1788 (27.4)
	Morphological anomalies	446 (6.8)
	Biological anomalies	11 (0.2)
	Particular clinical situations in a disease or syndrome	60 (0.9)
**Subtypes of disorders**		1084 (11.1)
	Clinical subtypes	839 (77.4)
	Etiological subtypes	195 (18)
	Histopathological subtypes	50 (4.6)
**Groups of disorders**		2173 (22.2)
	Clinical groups	450 (20.7)
	Categories	1723 (79.3)

^a^Percentages for top-level classification categories (Disorders, Subtypes of disorders, and Groups of disorders) are calculated relative to the total number of active clinical entities. Percentages for subordinate classifications are calculated relative to the total number within their respective parent category.

#### Counting and Distribution of English Synonyms Across Active Clinical Entities in the Orphanet Nomenclature of RDs

Of the 9784 active clinical entities in the Orphanet Nomenclature of RDs, 6311 (64.5%) carry at least 1 synonym in English, while the remaining 3473 (35.5%) are not associated with any synonym. Of the 6527 disorders, 4805 (73.6%) carry at least 1 synonym in English, with a total number of synonyms at the disorder level amounting to 10,547. The minimum number of synonyms associated with a disorder is 1, and the maximum is 15, with a mean of 2.2 (SD 1.6) synonyms for disorders with at least 1 synonym. The complete distribution of synonyms by classification level is reported in Multimedia Appendix 4.

#### Counting and Distribution of Translated Preferred Terms and Synonyms Across Translation Languages

All preferred terms associated with active clinical entities registered in the Orphanet Nomenclature of RDs are translated into the following languages: French, German, Spanish, Italian, Dutch, Portuguese, Polish, and Czech. Table 2 shows the rate of translation of preferred terms for every language. The very minor delay in translation is due to the different timings and frequencies at which translations are processed by every translation team. Nontranslated preferred terms are nevertheless included in English, pending translation. Synonyms can be adapted in all translated languages according to the medical practice in the target language; the number of adapted synonyms for every translation language is shown in Table 2. The complete distribution of synonyms by classification level in all translated languages is reported in Multimedia Appendix 4. Additional translations are also available in Chinese (9158 translated preferred terms, last update June 2020), Turkish (9168 translated preferred terms, last update September 2021), and Ukrainian (9612 translated preferred terms, last update July 2024), and are provided in separate files in the “rare diseases and alignment with terminologies and databases” section of Orphadata Science [46].

**Table 2:** Number of active clinical entities with translated preferred term and adapted synonyms by language.

Language	Translated preferred terms (n=9784), n (%)	Adapted synonyms, n
French	9784 (100)	13,000
German	9778 (99.9)	12,854
Spanish	9784 (100)	12,912
Italian	9569 (97.8)	11,234
Dutch	9777 (99.9)	14,546
Portuguese	9576 (97.9)	5858
Polish	9605 (98.2)	12,804
Czech	9628 (98.4)	14,298

#### Coverage and Distribution of Disorder Definitions Across Languages

Disorder definitions are produced in English for all disorders included in the Orphanet Nomenclature of RDs and are subsequently translated into the 8 translation languages: French, German, Spanish, Italian, Dutch, Portuguese, Polish, and Czech. Table 3 presents the coverage of disorder definitions at the disorder level in each language. Currently, 94% (6116/6527) of rare disorders have a definition in English, while the rate of coverage in other languages depends on the priorities defined by each translating country.

**Table 3:** Number of disorders with available definitions per language.

Language	Disorders with a definition (n=6527), n (%)
English	6116 (93.7)
French	5941 (91)
German	4572 (70.1)
Spanish	6095 (93.4)
Italian	4592 (70.4)
Dutch	6068 (92.9)
Portuguese	1186 (18.2)
Polish	1372 (21)
Czech	17 (0.26)

### Annual Inclusion and Inactivation of Disorders in the Orphanet Nomenclature of RDs Over the Past 6 Years (2020-2025)

Since 2020, an average of 123 (SD 72) new disorders per year have been added to the Orphanet Nomenclature, while an average of 44 (SD 18) disorders per year have been inactivated. A detailed report of the number of newly included, obsoleted, deprecated, and nonrare disorders for each year from 2020 to 2025 is provided in Table 4.

**Table 4:** Yearly overview of newly included and inactivated disorders in the Orphanet Nomenclature of Rare Diseases (2020-2025).

	Newly included disorders, n	Obsoleted disorders, n	Deprecated disorders, n	Nonrare disorders, n
2020	74	35	24	5
2021	53	8	16	1
2022	92	29	5	4
2023	114	21	8	5
2024	253	38	22	9
2025	152	13	19	2

### Distribution of Rare Disorders by Medical Domain in the Orphanet Classification of RDs

**Figure 3:**
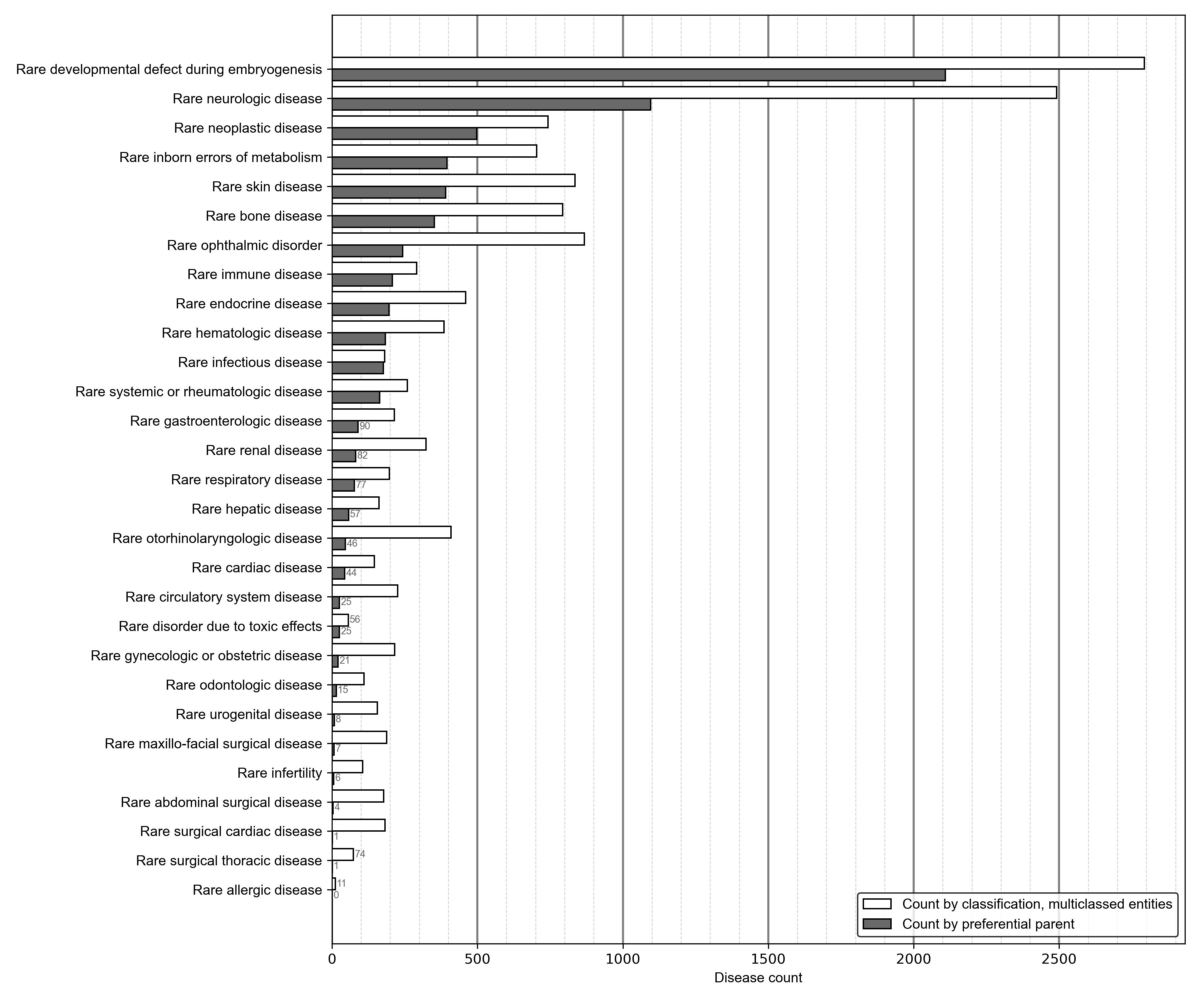
Distribution of rare disorders by medical domain according to their classification. Gray bars represent the number of disorders linearized according to their preferential parent classification, while white bars indicate the distribution of multiclassified disorders, counted as many times as they appear in the relevant classification hierarchies. Classifications containing fewer than 100 disorders are annotated with exact counts to enhance readability. The analysis includes 29 classification hierarchies corresponding to distinct medical domains; 5 hierarchies representing organizational or nonclinical groupings were excluded, as described in the Methods section. Six disorders were excluded from this analysis because they correspond to biological anomalies without clinical manifestations or were linearized under the classifications of rare genetic disorders or rare transplant-related disorders. ORPHA:616874 rare disorder without a determined diagnosis after full investigation was also excluded.

The Orphanet classification of RDs has a multiparental structure: 4415 (67.7%) disorders are classified in more than one classification hierarchy, while 2106 (32.3%) are classified in just one classification, independently of the number of parents that they have in this same hierarchy. Figure 3 illustrates this multiparental structure by comparing the number of disorders linearized to each medical domain and therefore counted once (gray bars), with the number of disorders classified in one or more medical domains and therefore counted as many times as they appear in each classification (white bars). As an example, while 1096 disorders are primarily classified as neurological disorders, a total of 2492 disorders present a major neurological involvement. Based on the linearization rules, the majority of rare disorders fall into the rare developmental defect during embryogenesis classification (n=2109, 32.3% disorders), closely followed by the rare neurologic disorders classification (n=1096, 16.8% disorders), amounting to almost 50% of all rare disorders. The following most-represented classifications are the rare neoplastic disorders at 7.6% (497/6527), the rare inborn errors of metabolism at 6.1% (395/6527), the rare skin disorders at 6% (391/6527), the rare bone disorders at 5.4% (352/6527), the rare ophthalmic disorders at 3.7% (243/6527), the rare immune disorders at 3.2% (208/6527), the rare endocrine disorders at 3% (196/6527), the rare hematologic disorders at 2.8% (184/6527), the rare infectious disorders at 2.7% (177/6527), and the rare systemic or rheumatologic diseases at 2.5% (164/6527). The remaining classifications each include fewer than 100 linearized disorders each, collectively representing the residual 8% (515/6527) of rare disorders (Figure 3 gray bars; Multimedia Appendix 5).

A similar pattern is observed when considering multiclassified disorders, where the largest numbers are again found in the rare developmental defect during embryogenesis classification (2793 disorders) and the rare neurologic disorders classification (2492 disorders). The following classifications, however, contain considerably fewer multiclassified disorders and are, in descending order: rare ophthalmic disorders (868 disorders), rare skin disorders (835 disorders), rare bone disorders (793 disorders), rare neoplastic disorders (743 disorders), and rare inborn errors of metabolism (704 disorders). All remaining hierarchies contain fewer than 500 multiclassified disorders each, with only 3 hierarchies containing fewer than 100 (Figure 3 white bars; Multimedia Appendix 5).

### Coverage and Proximity of the Mappings Between the Orphanet Nomenclature of RDs and Other Semantic Resources

Of the 6527 active disorders registered in Orphanet, 6355 (97.4%) are aligned to at least one code *ICD-10*, with only 415 (6.4% of all disorders) having an exact proximity relationship. The remaining aligned codes are either present in the *ICD-10* alphabetical index/are included in the tabulated list under the *ICD-10* code (655 disorders), or they have been attributed by Orphanet to the *ICD-10* code that corresponds to the closest entity according to Orphanet’s rules (Table 5). The work on *ICD-11* mapping has not been completed yet; however, as of July 2025, a total of 4683 (71.8%) disorders were mapped to at least one code in the *ICD-11* MMS, with 958 (14.7% of all disorders) characterized by an exact proximity relationship and 3013 (46.2%) present in the *ICD-11* Foundation with an exact Unique Resource Identifier. The remaining mappings have been attributed by Orphanet to the *ICD-11* code corresponding to the closest entity (Table 5).

**Table 5:** Number of active disorders mapped to other semantic resources.

	Active disorders with at least 1 mapping (n=6527), n (%)	Active disorders with an exact proximity relationship (n=6527), n (%)
*ICD-10* ^a^	6355^b^ (97.4)	415 (6.4)
*ICD-11* ^c^	4683^b^ (71.8)	958 (14.7)
SNOMED CT^d^	6191 (94.8)	6191 (94.8)
OMIM^e^	4141 (63.4)	3319 (50.9)
UMLS^f^	6501 (99.6)	6501 (99.6)
MeSH^g^	2735 (41.9)	2735 (41.9)
MedDRA^h^	1455 (22.3)	1455 (22.3)
GARD^i^	3279 (50.2)	3279 (50.2)
Mondo^j^	6194 (94.9)	6194 (94.9)

^a^ICD-10: International Statistical Classification of Diseases, Tenth Revision.

^b^Counts include mappings attributed by Orphanet when the rare disorder concept was not present in the target resource.

^c^ICD-11: International Classification of Diseases, Eleventh Revision.

^d^SNOMED CT: Systematized Nomenclature of Medicine Clinical Terms.

^e^OMIM: Online Mendelian Inheritance in Man.

^f^UMLS: Unified Medical Language System.

^g^MeSH: Medical Subject Headings.

^h^MedDRA: Medical Dictionary for Regulatory Activities.

^i^GARD: Genetic and Rare Diseases Information Center.

^j^Mondo: Mondo Disease Ontology.

According to the October 2025 release of the Orphanet-SNOMED CT mapping, 6191 (94.8%) active Orphanet disorders are mapped to 1 SNOMED CT code, all with an exact proximity relationship (Table 5).

Among all active disorders present in the Orphanet Nomenclature, 72.2% (n=4715) are genetic disorders. A total of 4141 Orphanet disorders are mapped to at least 1 phenotypic OMIM number, and 3319 mapped disorders have an exact proximity relationship with the corresponding phenotypic OMIM number (Table 5).

In addition, exact mappings to external semantic resources are distributed as follows: 6501 (99.6%) for the UMLS Metathesaurus, 2735 (41.9%) for MeSH, 1455 (22.3%) for MedDRA, 3279 (50.2%) for GARD, and 6194 (94.9%) for Mondo. Of note, only mappings to *ICD-10*, *ICD-11*, and OMIM are distributed together with the official Orphanet Nomenclature files for coding, while the entirety of the mappings is distributed through Orphadata Science.

## Discussion

### Understanding the Necessity of an RD-Specific Terminology

While collectively common, RDs are numerous, clinically heterogeneous, and share challenges directly related to their individual rarity: persons living with each RD are few and dispersed, knowledge about each RD is limited and often inaccessible to nonspecialist health professionals, and expertise is scarce and dispersed as well. RDs are hard to identify in medical records since most are absent from medical terminologies in use in hospitals or are not labeled as rare, rendering them invisible in health systems. This lack of identification results in inequalities in accessing a timely diagnosis and appropriate care, hinders data collection on RDs natural history and their individual and societal burdens, and complicates patient recruitment for clinical research and therapy development [4,8,17,49].

The availability of adequate codes for each RD is a well-recognized need at the international level [25]. Improved codification for RDs has been cited as a priority in the 2009 EU (European Union) Council Recommendation on an action in the field of RDs [27], and more recently by the Rare2030 recommendations [50]. Having codes for each RD would help international, European, national, and local health authorities obtain better knowledge of health care pathways and of the impact of RDs on both specialized (such as centers of expertise) and general health care services, while also supporting budget planning for health and social services [8,25,27]. Adequate coding is also necessary for identifying patients for clinical trials at both national and transnational levels. Finally, a large proportion of patients with RD remain undiagnosed despite undergoing a full investigation, but this particular situation cannot be captured by the codification systems in use.

RDs codification requires a nomenclature and classification system that meets the requirements of a standard medical terminology to ensure implementation in health information systems and in systematic research collections, such as patient registries. A trustworthy and reliable medical terminology must meet strict quality requirements: univocity and unambiguity of coded concepts, unrestricted management of synonyms, consistency of semantic relationships between concepts, multilingual support, accuracy of translations, correctness of semantic alignments, and relevant coverage of identified use cases. Additionally, terminology standards must adhere to rigorous and essential quality procedures, ensure sustainability and scalability to support the rapid development of knowledge, and guarantee long-term stability, preservation of the meaning of concepts from previous versions, and traceability of changes [51].

A key prerequisite for an RD terminology is to adopt a clear and operational definition of RDs. Orphanet adopts a clinical definition that encompasses all RDs, regardless of whether the etiology is known or not. This core concept ensures a terminology that can be adapted to different use cases, that is, health systems with diverse diagnostic capabilities or different stages of the patient’s diagnostic journey. An RD in Orphanet is therefore defined as a clinical entity characterized by a set of well-delineated phenotypic abnormalities, described in at least 2 independent cases in the medical literature, and a consistent evolution, allowing for a definitive clinical diagnosis. The rarity of a disease is defined according to the European prevalence threshold (not more than 1 in 2000 persons in the European population). This choice was made for historical reasons, as Orphanet was developed in Europe and cofunded by the European Commission (EC). However, this definition is consistent with those used in other geographical areas, which adopt either a similar or more restrictive cutoff, with the exception of the United States, where diseases with a higher prevalence are included [1,14]. Another limitation of this definition is that it includes infectious diseases that are rare in Europe, even if they are common in other parts of the world. To address this, some countries having adopted the Orphanet Nomenclature of RDs outside the European space, like Argentina, have adapted this chapter to exclude infectious diseases that are common in their countries [52]. These limitations underline the need for consensus around a global operational definition of RDs, a goal the WHO has worked toward in collaboration with RDI, with the participation of a multistakeholder, international group of experts, including an Orphanet representative (AR) [16]. The results of this work support Orphanet’s adoption of a clinical definition of RDs and may lead in the future to adaptations of the Orphanet RDs lists for the different WHO regions. In the meantime, Orphanet collaborates with non-European countries to help adapt the Orphanet Nomenclature of RDs to local needs, improving global RDs interoperability.

### Implications of the Orphanet Terminology Structure, Mappings, and Updates

The Orphanet Nomenclature and Classification of RDs is, to the best of our knowledge, the only RDs terminology clearly defining 3 levels of granularity: group of disorders, disorder, and subtype of disorders, with the disorder level complying with the internationally agreed-upon definition of RDs. This distinction has essential implications.

A first implication is that it allows for unambiguously counting the number of existing RDs (ie, the disorder classification level), preventing overcounting due to the aggregation of heterogeneous granularity levels. As of today, many enumerations of RDs circulate, some derived from semiautomated integration of different databases and ontologies (most frequently Orphanet and OMIM), sometimes resulting in estimates exceeding 10,000 RDs [8,53,54]. However, these methods aggregate different levels of granularity without clearly stating which RDs definition is adopted, rather reflecting the growing knowledge of disease mechanisms or treatable genetic variants than an actual increase in the number of RDs. While this overall figure aligns closely with Orphanet’s total active clinical entities (9784 as per the July 2025 release), it is essential to recognize that superimposing different levels of granularity and/or different etiologies of the same RDs can lead to counting the same disease multiple times. Using this broader count in public health discussions without clarification can lead to misinterpretations, potentially affecting policy decisions, budget allocation, and health care planning. For epidemiological and public health purposes, the count of clinically defined RDs (6527 according to the Orphanet July 2025 release) provides an appropriate common denominator for comparing countries and health care systems with varying technological and diagnostic capacities. It also permits to follow the evolution of therapy development and of health care resource allocation, including assessment of how many RDs benefit from these measures over time and across geographies. Conversely, for precision medicine or molecular diagnosis, a more granular approach, including subtypes (1084 in the July 2025 release) or information on genetic variants collected in patient records, may be needed.

A second implication is that defining the disorder granularity level allows the prevalence of RDs to be established accurately and facilitates comparability across countries and continents. A clear RDs definition, therefore, results in accuracy in counting RDs and, consequently, in counting patients, which is essential for assessing the magnitude of RDs as a public health issue. Furthermore, while allowing patient coding at the lowest granularity level (ie, subtypes of disorder), the classification hierarchies also allow data aggregation at the disorder level, thereby permitting to unambiguously establish the prevalence of RDs. Notably, the Orphanet Nomenclature of RDs is the only system that provides a code for undiagnosed patients with RD who underwent all available diagnostic possibilities. This population with RD has been estimated to represent up to 50% of all patients with RD, based on the experience of RDs expert centers [33]. Being able to code these patients at the disorder level makes it possible to include them in research pathways and undiagnosed programs, as recommended by the International Rare Diseases Research Consortium’s goal #1 [55-57], and to include them in future global RDs point prevalence estimations.

The 3-level classification structure of the Orphanet Nomenclature and Classification of RDs supports consistent semantic relationships between concepts, enabling effective use across various use cases and settings. While the disorder level serves as the primary reference level for data sharing among systems and countries, and for epidemiological purposes, groups of disorders allow data aggregation for use cases analysis, that is, for public health decision-making, such as health care organizations (eg, designation of centers of expertise by RDs area). Meanwhile, subtypes of disorders allow for fine-tuning of RDs codification at the clinical, etiological, and histopathological level, and can be used for applications in genetics, precision medicine, and other research and innovation purposes [58-64].

The Orphanet classification system is also able to capture the multisystemic nature of RDs, as clinical entities are organized into 29 distinct medical domains and classified as many times as needed according to their relevant domain of care. This is reflected in the distribution of RDs across classification hierarchies: the majority of RDs fall within the rare developmental defect during embryogenesis and rare neurologic disease classifications, together accounting for nearly 50% of all RDs. However, almost 70% of RDs are classified in more than one classification, reflecting their multisystem involvement. This approach ensures a more accurate representation of the complex nature of RDs and of their linked resources, allowing for better orientation of patients and medical doctors toward appropriate information and specialized care [58-60]. At the same time, the distribution of entities across preferential parent classifications is uneven, with some domains encompassing a large proportion of RDs and others including comparatively fewer entities. This distribution has implications for statistical analysis and data representation, as domains with fewer entities may appear less represented when preferential parent classifications are used for data aggregation. However, the multihierarchical structure allows flexible aggregation strategies that can be tailored to specific epidemiological or public health questions.

The field of RDs is a rapidly evolving one: with the continuous advancement in genetic knowledge and the growing interest in personalized medicine, new RDs are described each month. Additionally, the scope of previously identified RDs can be refined, with medical concepts being either merged or divided based on new discoveries or the examination of larger numbers of patients. To ensure that the Orphanet Nomenclature and Classification of RDs remain up to date with current scientific knowledge, Orphanet conducts a monthly systematized review of the literature and continuously collaborates with medical experts, particularly from the European Reference Networks and international RDs learned societies [32,65]. Decisions on modifications to ORPHAcodes are made after an extensive review of the literature, and are validated by the Orphanet medical and scientific committee, following published standardized procedures [29]. Between 2020 and 2025, a total of 738 new RDs were included in the Orphanet Nomenclature of RDs, averaging about 120 RDs per year. This number reflects not only newly described RDs, but also the outcomes of numerous time-effective collaborative projects with experts, aimed at restructuring and updating the classification to incorporate revised medical concepts. At the same time, Orphanet also applies a strict inactivation policy to preserve the stability and univocity of ORPHAcodes. Inactivated codes are never reused for other entities, and a substitution code is always suggested to maintain traceability. While unusual, it is possible to reintroduce an inactivated ORPHAcode when new evidence emerges that justifies a reconsideration [29]. This has occurred in cases such as systemic lupus erythematosus ORPHA:536 and immunoglobulin A nephropathy ORPHA:34145, which did not initially meet RDs prevalence criteria and are now considered RDs. Taken together, the integration of new RDs and the number of inactivated ones contribute to a small and relatively stable annual increase in the total number of RDs, averaging fewer than 80 RDs per year. A small proportion of active clinical entities are identified as historic, corresponding to RDs for which no new patients have been reported in the medical literature for 25 years. These entities are retained in the nomenclature to preserve diagnoses that could potentially reemerge and to support retrospective analyses, while their specific status limits the risk of confusion.

To further ensure the univocity and accessibility of concepts, Orphanet implements an unrestricted management of synonyms. Synonyms must cover the exact scope of the concept and are added as needed, without limitations in number [29]. This approach maximizes semantic coverage across different usages and data sources. Additionally, Orphanet provides a standardized, medically validated definition for each RD included in the Orphanet Nomenclature. These definitions consist of short texts describing the clinical features to unambiguously delineate the entity and distinguish it from other RDs, and are intended to support health care professionals in assigning the correct ORPHAcode to a patient [29,66]. To further ensure international interoperability, the Orphanet Nomenclature and Classification of RDs is also a multilingual resource. Translations are performed according to established procedures that include a final manual medical validation [31]. The different translations also include an unlimited number of synonyms that are tailored according to the relevance for each language. Taken together, these elements ensure semantic consistency and facilitate ORPHAcode integration into health and research information systems.

RDs is a field in which the boundaries between diagnosis, health care, and research are often blurred; maintaining semantic interoperability between health and research sectors is essential. The multiplicity of reference semantic standards used in health care, research, and regulatory science reflects the fact that each serves a distinct purpose, adopting diverse levels of granularity and encoding clinical concepts according to its own organizing principles. For example, *ICD* classifications are optimized for morbidity and mortality statistics, billing and reimbursement, and administrative reporting, which often results in broad disease groupings. In contrast, research-oriented resources such as OMIM organize information around gene-phenotype relationships, frequently splitting a single clinically defined disease into multiple molecular entries or, on the contrary, merging distinct clinical disorders related to the same gene. Consequently, semantic alignments between terminologies entail inherent complexity and need domain expertise, and must not be represented as simple one-to-one correspondences, but rather as structured relationships, as described in studies investigating RDs terminology harmonization and mapping processes [67]. Mapping strategies must therefore explicitly document mapping cardinality and capture varying degrees of conceptual proximity and specificity, in order to preserve meaning across contexts and avoid misinterpretation. In addition, while automated approaches may assist in identifying candidate correspondences, the validation of RDs mappings requires expert medical review to ensure that assigned relationships accurately reflect clinical meaning. This is reflected in the way ORPHAcodes are mapped to different resources, with defined relationships and medical validation. As of July 2025, Orphanet has aligned 97.4% (6355/6527) of its active rare disorders to at least 1 *ICD-10* code, with only 6.4% (415/6527) of all RDs being exactly represented. The work on *ICD-11* mapping is ongoing, but by July 2025, 71.8% (4683/6527) of Orphanet’s RDs were mapped to at least 1 *ICD-11* MMS code, with 14.7% (958/6527) of all RDs being exactly represented. Additionally, the October 2025 release of the Orphanet-SNOMED CT mapping shows that 94.8% (6191/6527) of Orphanet’s active RDs are mapped to SNOMED CT, with all exhibiting an exact proximity relationship. Regarding genetic diseases, Orphanet classifies 4715 (72.2% of active disorders) RDs as genetic (defined as either caused by a known gene or having a clear familial inheritance pattern), with 4141 RDs mapped to one or more phenotypic OMIM numbers. It is interesting to note that many genetic RDs are mapped to multiple OMIM numbers, reflecting the fact that Orphanet adopts a clinical definition of disease, whereas OMIM organizes entries around individual gene-phenotype relationships.

Orphanet is currently the only primary producer of a scientifically validated nomenclature and classification of RDs. Other terminologies are increasingly integrating Orphanet concepts as a source of RDs information, acknowledging the critical scientific contribution Orphanet makes to the RDs community. Outstanding collaborations between Orphanet, SNOMED International, and WHO ensure that the RDs content of SNOMED CT and the *ICD-11* continue to be improved by the inclusion of RDs concepts as defined by Orphanet [23,24].

In parallel with mappings to major reference standards, the alignment of ORPHAcodes with other semantic resources, including UMLS, MeSH, MedDRA, GARD, and Mondo, supports a range of use cases such as literature indexing, pharmacovigilance, regulatory reporting, and knowledge integration. Overall, this extensive and accurate mapping system ensures consistency of transcoding across multiple platforms, further enhancing the use of the Orphanet Nomenclature of RDs in clinical, research, and regulatory contexts [61-64,68,69].

### Recognition and Adoption of the Orphanet Nomenclature of RDs

Since 1997, Orphanet has maintained through its publicly available website an inventory of RDs to provide information on RDs and their dedicated services across Europe [28]. This has evolved over time into a nomenclature and classification system of RDs, which is today the only RDs-specific standard meeting the high-quality requirements of a medical terminology [20].

In 2014, the EC Expert Group on Rare Diseases adopted a recommendation on improving RDs codification [25]. In this document, EU Member States are encouraged to assess the feasibility of implementing ORPHAcodes at a national level and to include RDs codification into their national RDs plans/strategies. In 2017, the EC Steering Group on Promotion and Prevention recognized ORPHAcodes as a best practice [70]. Since then, ORPHAcodes have been included in the set of common data elements for Rare Diseases Registration, released by the EU RDs platform on RDs registration to enhance interoperability between registries [71]. The Orphanet Nomenclature of RDs has also played a key role in implementing the EU 2011 Cross-Border Healthcare Directive in regard to RDs. As a result, ORPHAcodes have been recommended for tracking RDs diagnoses in the European Common Semantic Strategy (2019) [72] and the Guidelines on Patient Summary (release 3.2, March 2022, and release 3.4, November 2024) [73], and have been integrated into the code systems used in the Master Value Sets Catalogue—My Health@EU—eHealth Digital Service Infrastructure [74,75]. The functional specifications developed by the X-eHealth project were instrumental for including ORPHAcodes in the minimum dataset required for the electronic patient summary [73,76]. Finally, the World Health Assembly resolution “Rare diseases: a global health priority for equity and inclusion,” adopted on May 24, 2025, urges member states to commit to “considering the implementation of *ICD-11*, and where appropriate, interoperable codification systems for RDs such as the Orphanet Nomenclature of RDs, at their earliest possibility, and in accordance with their available resources, in order to enable the recording, reporting and monitoring of rare diseases at the national and international levels” [77].

To support the effective and sustainable implementation in health and research information systems, the Orphanet Nomenclature of RDs (ORPHAcodes) is distributed under an open license (CC-BY 4.0) on different platforms depending on use: Orphadata Science for structured datasets intended for large-scale analyses [46], and the Orphacode.org platform [78] for coding resources for health information systems [47]. Each annual release (in July of each year) is versioned and accompanied by differential files to ensure traceability of changes and continuity of coding, as per the recommendation of the RD-ACTION working group for routine maintenance of codification [79]. In addition, a suite of technical tools is provided to facilitate implementation into diverse systems, promote accurate patient codification, and ensure continuous support by the community. Finally, the implementation of ORPHAcodes within information systems should be guided by established and evolving guidelines and best-practice recommendations, including those developed within the RD-CODE and the OD4RD/OD4RD2 project [33,80-82]. This supporting infrastructure ecosystem is essential to ensure evolution over time and respond to emerging use cases in a rapidly evolving field.

Establishing a seamless data ecosystem for RDs based on the Orphanet Nomenclature and Classification of RDs is a key step toward increasing the knowledge in a field that has long remained largely invisible within health systems, and ultimately improving the lives of people living with RD [50]. Indeed, accurate codification of patients with RD enables the production of data for both primary and secondary use. Primary use includes, among others: recognition of the RDs condition by nonexpert health professionals, ensuring best care practices at all stages of the patient’s care pathway, and recognition of undiagnosed patients with RD who might benefit from new discoveries, potentially leading to establishing a diagnosis. Secondary use of RDs data includes accurately measuring the prevalence of the entire population with RD and monitoring public health interventions to inform evidence-based decision-making, producing real-world evidence for research and therapeutic development, and facilitating recruitment for clinical research.

### Conclusions

The Orphanet Nomenclature and Classification of RDs is the only RD-specific, high-quality standard, interoperable medical terminology meeting the needs of health care, research, and public health systems. By providing a clinically based and scientifically validated framework, it helps close the RDs visibility gap in health and research information systems, enabling consistent RDs identification and monitoring. Its wide adoption will support cross-border and cross-sectoral data interoperability, contributing to improved knowledge, policymaking, and ultimately better care for people living with a RD.

## Data Availability

The datasets supporting the conclusions of this study are publicly available. Orphanet provides open access to rare disease nomenclature, classification, genetic data, and mappings to other terminologies [28]. Structured datasets for large-scale analysis are distributed via the Orphadata Science platform [46], and all previous versions are archived in a dedicated GitHub repository [83]. Additional resources include the Orphanet Nomenclature files for coding (Nomenclature Pack) distributed on the ORPHAcodes platform [78] and the Orphanet Rare Disease Ontology [84]. All formats are maintained and updated annually or biannually to ensure accuracy and accessibility.

## Appendix

Multimedia Appendix 1 Glossary of key concepts and definitions used in the Orphanet Nomenclature and Classification of Rare Diseases.

Multimedia Appendix 2 Overview of the 34 Orphanet classification hierarchies and their status as distinct medical domains or organizational classifications.

Multimedia Appendix 3 Distribution of Orphanet clinical entities by classification level and typology. This file is the output of Notebook 1_number_of_Orphanet_clinical_entities.

Multimedia Appendix 4 Production of the Orphanet RD terminology in English and translation languages. This file is the output of Notebook 2_production_of_the_Orphanet_RD_terminology_in_english_and_other_languages.

Multimedia Appendix 5 Distribution of disorders in Orphanet classifications according to their preferential parent and multiclassified disorders. This file combines the outputs of Notebook 5_distribution_of_Orphanet_RD_in_Orphanet_classifications and Notebook 6_distribution_of_Orphanet_RD_by_preferential_parent.

## Abbreviations

EC: European Commission
EU: European Union
GARD: Genetic and Rare Diseases Information Center
ICD: International Classification of Diseases
ICD-10: International Statistical Classification of Diseases, Tenth Revision
ICD-11: International Classification of Diseases, Eleventh Revision
INSERM: Institut national de la santé et de la recherche médicale
MedDRA: Medical Dictionary for Regulatory Activities
MeSH: Medical Subject Headings
MMS: Mortality and Morbidity Statistics
Mondo: Mondo Disease Ontology
NIH: National Institutes of Health
OMIM: Online Mendelian Inheritance in Man
RD: rare disease
RDI: Rare Diseases International
SNOMED CT: Systematized Nomenclature of Medicine Clinical Terms
UMLS: Unified Medical Language System
WHO: World Health Organization
